# Supplementary material for: Bioaccumulation of Lanthanum by Two Strains of Marine Diatoms Nanofrustulum shiloi and Halamphora kolbei
Source: Biology (Basel). 2025 Oct 24;14(11):1489. doi: 10.3390/biology14111489 (PMC12650198; doi:10.3390/biology14111489)
Supplement: Supplementary file 1 [file biology-14-01489-s001.zip › biology-3917331-supplementary.pdf]

Supplementary Material

Table S1. Results of the two-way PERMANOVA test for diatom abundance in control and experimental cultures at different La concentrations in the nutrient medium throughout the exposure period.

Strain / Concentration	10 mg La·l^{-1}		50 mg La·l^{-1}	
	F	*p*	*F*	*p*
Nanofrustulum shiloi	4.2252	0.0001	22.081	0.0001
Halamphora kolbei	2.3281	0.0027	10.959	0.0001

Table S2. Mean lanthanum content on the surfaces of diatom frustules and its variation range at the at low La concentration (10 mg·l^{-1}) in the nutrient medium.

Exposure time	*Nanofrustulum shiloi*		*Halamphora kolbei*	
	**Mean weight fraction, %**	**Weight fraction variation range, %**	**Mean weight fraction, %**	**Weight fraction variation range, %**
1 h	0.11	0–0.4	0.25	0.2–0.3
3 h	0.39	0–1.5	0.60	0.3–0.8
6 h	0.37	0–1	0.27	0.2–0.3
1 day	0.73	0.2–2.1	3.02	1.3–4.2
3 days	1.16	0–4.4	5.15	1.9–10.2
6 days	0.91	0.1–2.5	3.46	0–6.6
9 days	0.77	0–2.8	1.63	0–5.2
12 days	0.50	0–4.8	1.97	0.5–4

Table S3. Average lanthanum content on the surfaces of diatom frustules and its range of variation at the element concentration of 50 mg·l^{-1} in the nutrient medium.

Exposure time	*Nanofrustulum shiloi*		*Halamphora kolbei*	
	**Mean weight fraction, %**	**Weight fraction variation range, %**	**Mean weight fraction, %**	**Weight fraction variation range, %**
1 h	0.01	0–0.1	0	0
3 h	0	0	0	0
6 h	0	0	0	0
1 day	0.02	0–0.1	0.06	0–0.1
3 day	0.07	0–0.1	0.02	0–0.2
6 day	0.35	0–0.7	0	0
9 day	1.50	0.7–3.5	0.07	0–0.2
20 day	0.50	0–1.1	-	-

Table S4. Element concentrations (μg·g^{-1}) in the biomass of diatoms cultivated in the La-supplemented (10 mg La ·l^{-1}) and control (C) media.

Element	*Halamphora kolbei* La	*Halamphora kolbei* C	*N. shiloi* La	*N. shiloi* C
Be	0.007	0.006	0.008	0.006
B	1027	1162	1340	1249
Na	9530	20685	16154	23277
Mg	10971	10539	13843	12914
Al	371	428	628	481
P	4184	10467	4187	9948
S	1388	2542	2078	2832
K	2734	9917	6910	8424
Ca	923	3360	1396	5318
V	0.80	0.40	1.05	0.55
Cr	2.428	2.836	2.837	2.866
Fe	1309	780	1373	892
Mn	22.0	109	24.7	102
Fe	2129	1291	2237	1485
Co	0.57	0.44	0.70	0.53
Ni	7.25	0.26	1.80	0.33
Cu	11.4	6.98	7.98	6.45
Zn	22.5	20.8	22.1	20.5
Ga	0.30	0.31	0.31	0.35
As	1.17	1.22	1.83	1.58
Se	172	0.30	204	0.15
Rb	1.57	2.74	3.29	2.67
Sr	95.2	257	145	470
Mo	835	1300	2018	1266
Ag	3.25	7.34	1.96	9.39
Cd	0.14	0.14	0.10	0.10
Cs	0.014	0.016	0.020	0.012
Ba	8.54	27.2	9.39	25.5
La	6065	3.08	6906	7.07
Ce	6.60	0.46	6.73	0.45
Pr	0.038	0.048	0.067	0.051
Nd	0.18	0.16	0.23	0.18
Sm	0.037	0.024	0.034	0.033
Eu	0.005	0.005	0.003	0.007
Gd	0.519	0.028	0.594	0.032

Dy	0.003	0.024	0.025	0.028
Ho	0.003	0.005	0.005	0.005
Er	0.036	0.027	0.062	0.028
Tm	0.003	0.003	0.004	0.004
Yb	0.003	0.020	0.025	0.024
Lu	0.003	0.004	0.004	0.004
Tl	0.003	0.006	0.008	0.007
Pb	5.46	2.17	2.00	1.77
Th	0.092	0.057	0.006	0.061
U	0.956	0.096	0.387	0.216
